# Editorial: Immune checkpoints regulatory mechanisms and immunotherapy strategies in gastrointestinal tumors

**DOI:** 10.3389/fonc.2025.1697974

**Published:** 2025-09-24

**Authors:** Qing Xi, Rongxin Zhang

**Affiliations:** ^1^ Department of Clinical Laboratory, National Center for Respiratory Medicine, National Clinical Research Center for Respiratory Disease, State Key Laboratory of Respiratory Disease, Guangzhou Institute of Respiratory Health, The First Affiliated Hospital of Guangzhou Medical University, Guangzhou, China; ^2^ Laboratory of Immunology and Inflammation, School of Life Sciences and Biopharmaceutics, Guangdong Pharmaceutical University, Guangzhou, China

**Keywords:** immune checkpoints, PD-L1, PD-1, immunotherapy, gastrointestinal cancers

The Research Topic focuses on the rapidly evolving field of immune checkpoint regulation mechanisms and immunotherapy strategies in gastrointestinal tumors. It compiles ten representative articles, such as research papers, case reports, systematic reviews, and reviews, highlighting the current research progress and clinical challenges in this field from diverse perspectives. These achievements not only cover various gastrointestinal malignancies such as colorectal cancer, gastric cancer, esophageal cancer, pancreatic cancer, and rare liver tumors, but also delve into key scientific issues, including the predictive value of molecular biomarkers, optimization of combination therapy strategies, and regulation mechanisms of the tumor microenvironment. This reflects the translational medical research approach from basic science to clinical practice.

The case reports demonstrate that immunotherapy has therapeutic potential in traditionally high-risk or special populations, such as organ transplant recipients, and in rare pathological types of tumors, such as metastatic small bowel adenocarcinoma, hepatic adenosquamous carcinoma, and colon squamous cell carcinoma. These cases highlight the critical role of biomarkers such as dMMR in guiding immunotherapy, and also emphasize the value of multidisciplinary team (MDT) approaches and individualized treatment strategies in complex clinical practice.

Five original studies have further advanced our understanding of immune checkpoint regulation by elucidating underlying mechanisms. For instance, IL-6 has been identified as a negative biomarker for anti-PD-1 therapy response in esophageal squamous cell carcinoma, with its high expression closely associated with an immunosuppressive microenvironment; CXCL13 combined with anti-PD-1 treatment can delay tumor growth *in vivo*, significantly enhancing the immunotherapy response in gastric cancer by recruiting CXCR5^+^CD8^+^ T cells; CXCR2P1, as a novel hub gene, can influence antigen presentation and T cell activation by regulating MIR215, thereby enhancing sensitivity to PD-1 inhibitors. Moreover, combination therapy strategies such as pembrolizumab combined with chemotherapy have significantly improved survival in MSI-H/dMMR metastatic colorectal cancer. The combination of oxaliplatin with anti-PD-1 can synergistically inhibit the progression of colorectal cancer and alter the tumor immune microenvironment, thereby enhancing anti-tumor immunity. These studies provide new directions for overcoming current immune resistance.

The systematic review indicate that in pancreatic cancer, a “cold tumor,” immune checkpoint inhibitors combined with chemotherapy have demonstrated modest synergistic effects, but their combination with radiotherapy requires more cautious patient selection, indicating that different strategies may yield distinct biological effects and clinical outcomes. Combination strategies should not only consider mechanism complementarity but also balance efficacy and safety.

The review on PIK3CA mutations systematically summarizes the impact of PIK3CA gene mutations on targeted therapy and immunotherapy in colorectal cancer, as well as their potential value and future directions in personalized treatment. It highlights its potential clinical utility while recognizing existing challenges, such as insufficient standardization of detection, unclear functional mechanisms, unknown impact mechanisms on colorectal cancer treatment, and a lack of clinical validation. More clinical trials and evidence are needed to verify its efficacy and safety, as well as to optimize its treatment protocols and guidelines.

Collectively, the studies in this Research Topic point to a central theme: the future of immunotherapy for gastrointestinal tumors will depend upon in-depth molecular research and multi-omics technologies to establish precise biomarker systems and optimize individualized combination strategies. Such approaches will be essential for overcoming current therapeutic limitations.

